# Fungi–Bacteria Correlation in Alcoholic Hepatitis Patients

**DOI:** 10.3390/toxins13020143

**Published:** 2021-02-14

**Authors:** Bei Gao, Xinlian Zhang, Bernd Schnabl

**Affiliations:** 1 School of Marine Sciences, Nanjing University of Information Science and Technology, Nanjing 210044, China; wintergb@hotmail.com; 2 Department of Medicine, University of California San Diego, La Jolla, CA 92093, USA; 3 Division of Biostatistics and Bioinformatics, Department of Family Medicine and Public Health, University of California San Diego, San Diego, CA 92093, USA; xizhang@health.ucsd.edu; 4 Department of Medicine, VA San Diego Healthcare System, San Diego, CA 92161, USA

**Keywords:** mycobiota, ITS sequencing, alcoholic hepatitis

## Abstract

Alcohol-related liver disease is one of the most prevalent types of chronic liver diseases globally. Alcohol-related liver disease begins with fatty liver, which further develops into hepatic inflammation, hepatocyte injury, and progresses to fibrosis and cirrhosis. Compositional changes of gut bacteria and fungi were found in patients with alcohol-related liver disease. However, the functional changes of fungi and correlations between fungi and bacteria have not been investigated. In this study, we first examined the functional capacity of fungi in patients with alcohol-related liver disease using shotgun metagenomics. Among 24 MetaCyc pathways contributed by fungi, superpathway of allantoin degradation in yeast was enriched in patients with alcoholic hepatitis. Furthermore, we compared the predictive power of bacteria versus fungi and found that bacteria performed better than fungi to separate patients with alcoholic hepatitis from non-alcoholic controls and patients with alcohol use disorder. Finally, we investigated the associations between the intestinal fungi and bacteria in alcoholic hepatitis patients. Positive association between fungi and bacteria was found between *Cladosporium* and *Gemmiger*, meanwhile negative association was found between *Cryptococcus* and *Pseudomonas* in alcoholic hepatitis patients.

## 1. Introduction

The human gut microbiota consists of bacteria, eukaryotes, archaea, and viruses, which live symbiotically in the gastrointestinal tract [1]. The gut microbiota plays a key role in human health and disease. Alcohol-related liver disease is one of the most prevalent liver diseases worldwide. Alcohol-related liver disease progresses from simple steatosis to steatohepatitis, fibrosis, and cirrhosis. Alcoholic hepatitis is a severe form of alcohol-related liver disease and most commonly arises acutely on the background of cirrhosis [2]. Treatment strategies are limited for patients with alcoholic hepatitis. Pronounced overgrowth of bacteria in the small intestine was found in patients with alcoholic hepatitis [3]. Changes in the fecal bacteria were associated with disease severity in patients with alcoholic hepatitis [4]. 

In addition to bacteria, intestinal mycobiota was also dysregulated in patients with alcoholic hepatitis as revealed by fungal-specific internal transcribed spacer (ITS) amplicon sequencing [5]. Detailed taxonomic information could be revealed by this approach, however, the functional analysis of mycobiota is lacking. Shotgun metagenomics could provide information on the functional capacity of mycobiota. Although both bacteria and fungi are dysregulated in alcoholic hepatitis, previous efforts have been focused on either gut bacteria or fungi. The relationship between fungi and bacteria living in the same gastrointestinal tract is not well studied, although fungi and bacteria not only evolve together but also compete for resources. 

Antibiotics are commonly used in alcoholic hepatitis patients for the treatment of infections. However, it is not clear whether fungi would overgrow when the gut bacteria are impacted by the antibiotic treatment. Although such studies in humans are lacking, commensal bacteria were reported to inhibit the colonization of *Candida albicans* through the activation of hypoxia-inducible factor 1 α and antimicrobial peptide LL-37 in mice [6]. This raises the question whether changes of bacteria induced by antibiotic treatment in alcoholic hepatitis patients would further influence the growth of fungi, which is important in clinical practice. In addition, human gut Bacteroidetes can utilize yeast mannan via the expression of a specific yeast α-mannan degrading system [7]. Administration of *Saccharomyces boulardii* altered the gut microbial composition [8]. These findings provide evidence for bacteria–fungi interactions, which requires attention when developing gut microbiota-based therapeutic strategies, as targeting bacteria only or fungi only might also influence other parts of the intestinal microbiota in patients with alcoholic hepatitis.

Here, we sought to reveal the correlation between intestinal fungi and bacteria in patients with alcoholic hepatitis. Better understanding of the relationship between intestinal fungi and bacteria is helpful for the development of gut microbiota-based therapeutic strategies for alcoholic hepatitis patients, which not only take bacteria into consideration during the treatment but also fungi.

## 2. Results

### 2.1. Patient Characteristics

Patient characteristics are summarized in Table 1 and Table 2. The median age was higher in patients with alcoholic hepatitis, in comparison to patients with alcohol use disorder. Body mass index was higher in patients with alcoholic hepatitis compared with patients with alcohol use disorder. Patients with alcoholic hepatitis showed lower liver synthesis parameters and elevated liver function tests compared with patients with alcohol use disorder. Liver biopsy was available from 58% of the patients with alcoholic hepatitis, with 71% of these patients showing cirrhosis (Table 2).

### 2.2. Percentage of Bacteria and Fungi in Alcoholic Hepatitis Patients

**Figure 1:**
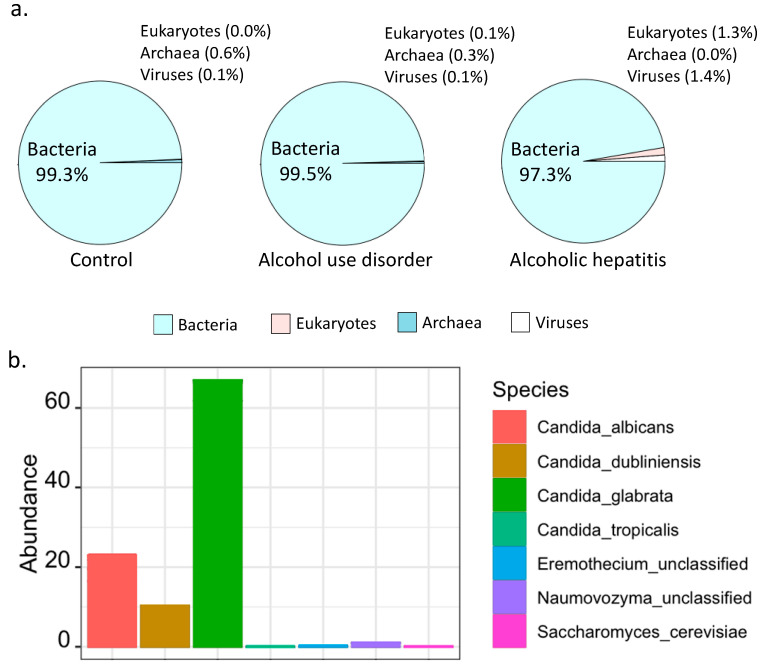
Fungal species and pathways revealed by shotgun metagenomics. (**a**) Microbial composition at kingdom level detected in control subjects (left, *n* = 9), alcohol use disorder patients (middle, *n* = 41), and alcoholic hepatitis patients (right, *n* = 81). (**b**) Fungal species in alcoholic hepatitis patients.

**Figure 2:**
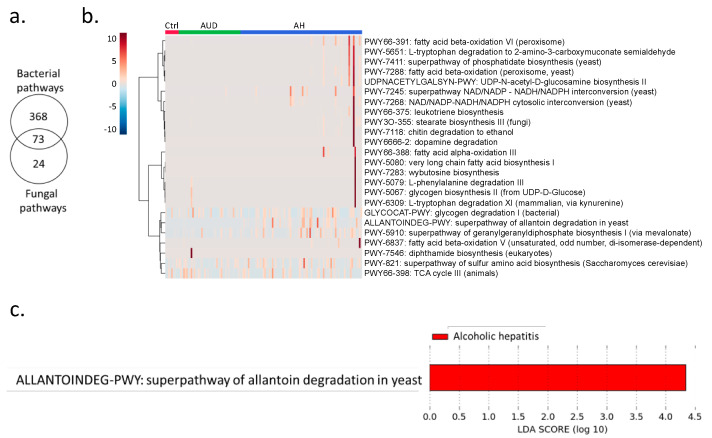
(**a**) Number of detected MetaCyc pathways contributed by fungi and bacteria. (**b**) MetaCyc pathways contributed by fungi. (**c**) Enriched fungal pathways in alcoholic hepatitis patients. LDA: linear discriminant analysis.

Shotgun metagenomics analysis showed that bacteria were predominant in controls, patients with alcohol use disorder and alcoholic hepatitis (Figure 1a). Notably, the percentage of kingdom eukaryote slightly increased in alcoholic hepatitis patients together with a decreased percentage of kingdom bacteria (Figure 1a). Seven fungal species were detected in stool samples by shotgun metagenomics sequencing, among which *Candida glabrata* was the most abundant species (Figure 1b). A total of 465 microbial pathways were detected in our patient cohort including non-alcoholic controls, patients with alcohol use disorder, and patients with alcoholic hepatitis, among which 368 were contributed by bacteria alone, 73 were contributed by both bacteria and fungi, while 24 pathways were contributed by fungi alone (Figure 2a,b). Among the 24 fungal pathways, ALLANTOINDEG-PWY: superpathway of allantoin degradation in yeast, was enriched in patients with alcoholic hepatitis (Figure 2c). 

### 2.3. Prediction of Alcoholic Hepatitis

**Figure 3:**
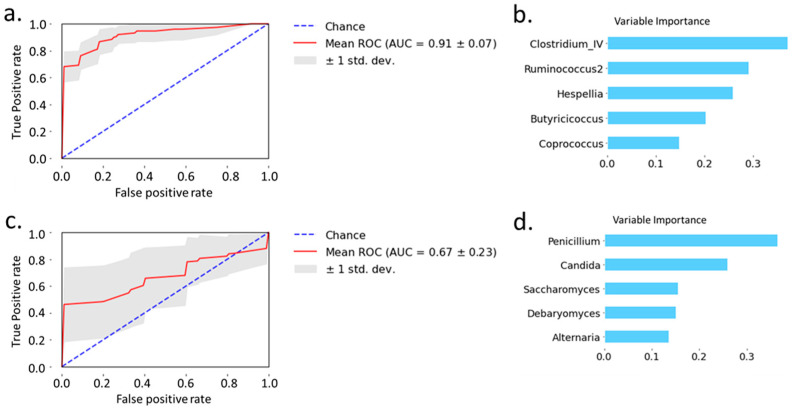
Prediction of alcoholic hepatitis patients from non-alcoholic controls and patients with alcohol use disorder using random forest model. (**a**) Prediction of alcoholic hepatitis patients using bacteria. (**b**) Variable importance of bacteria. (**c**) Prediction of alcoholic hepatitis using fungi. (**d**) Variable importance of fungi.

Next, we examined the predictive power of bacteria and fungi, respectively, to separate patients with alcoholic hepatitis from non-alcoholic controls and patients with alcohol use disorder. Random forest analysis showed that the area under the curve achieved 0.91 when using 5 selected bacteria to distinguish alcoholic hepatitis patients from non-alcoholic controls and patients with alcohol use disorder (Figure 3a). The variable importance is shown in Figure 3b. Random forest analysis of 5 selected fungi achieved an area under the curve of 0.67 when distinguishing alcoholic hepatitis patients from non-alcoholic controls and patients with alcohol use disorder (Figure 3c). The variable importance for fungi is shown in Figure 3d. Taken together, bacteria showed better predictive power than fungi when distinguishing patients with alcoholic hepatitis from non-alcoholic controls and patients with alcohol use disorder.

### 2.4. Fungi–Bacteria Network

**Figure 4:**
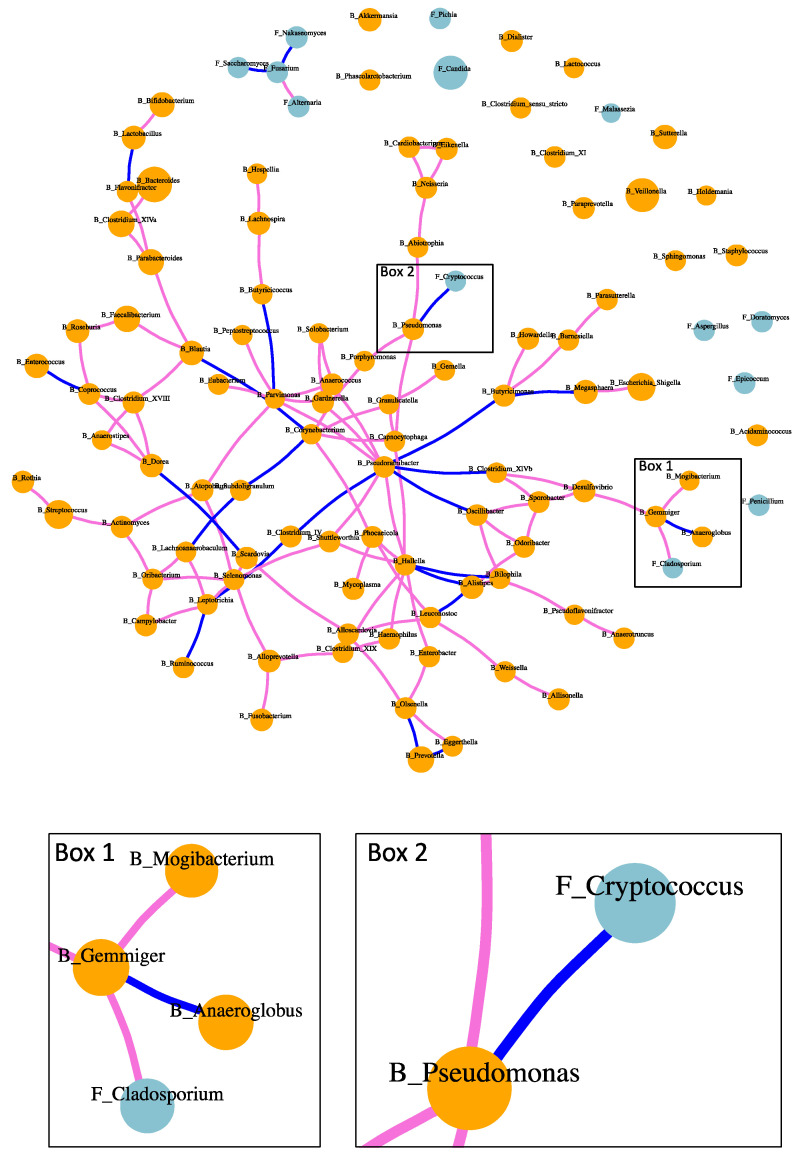
Fungi–bacteria network in patients with alcoholic hepatitis. Orange dots: fungi; orchid edge: positive correlation; blue edge: negative correlation.

Both positive and negative associations were found between intestinal fungi and bacteria in patients with alcoholic hepatitis (Figure 4). Positive association between fungi and bacteria was found between *Cladosporium* and *Gemmiger* (Figure 4 Box 1). Negative association between fungi and bacteria included *Cryptococcus* and *Pseudomonas* (Figure 4 Box 2). Positive and negative correlations were also found between bacteria and bacteria, as well as fungi and fungi (Figure 4). For instance, bacterium *Pseudoramibacter* was positively correlated with five bacterial genera, while negatively associated with four bacteria genera. Fungus *Fusarium* was positively correlated with fungus *Alternaria,* while negatively correlated with fungi *Saccharomyces* and *Nakaseomyces*. 

## 3. Discussion

In the present study, we found an altered fungal pathway in patients with alcoholic hepatitis, predicted the diagnosis of alcoholic hepatitis using random forest model, and identified the association between fungi and bacteria in patients with alcoholic hepatitis. Superpathway of allantoin degradation in yeast was enriched in alcoholic hepatitis (Figure 2c). Allantoin is a product of guanine and adenine catabolism, which is a product of uric acid oxidation and can serve as a sole nitrogen source and be degraded to ammonia [9]. Allantoin can be used by both fungi and bacteria as carbon and nitrogen sources. However, the pathway for conversion of uric acid to allantoin has been lost in humans. Elevated uric acid has been reported to contribute to experimental and human alcohol-related liver disease [10]. The elevation of uric acid might lead to the enrichment of allantoin degradation in the gut microbiota. However, the potential contribution of allantoin degradation to the pathogenesis of alcohol-related liver disease warrants further analysis.

Fungi and bacteria share micro-habitats and engage in complex communications within the microbial community. Such relationship cannot be easily predicted based on the knowledge of cultured microorganisms, especially in the disease condition of alcoholic hepatitis. The metabolic crosstalk develops interdependently, which leads to co-occurrence patterns of fungi and bacteria. The correlation network of fungi and bacteria in our study revealed these co-occurrence patterns in patients with alcoholic hepatitis. Especially, a negative association was found between fungus *Cryptococcus* and bacterium *Pseudomonas.* Species in *Pseudomonas* such as *Pseudomonas aeruginosa* are also responsible for life-threatening infections in immunocompromised persons [11]. Since negative correlation exists between fungus *Cryptococcus* and bacterium *Pseudomonas,* treatment which targets the growth of *Pseudomonas* might increase the growth opportunity for *Cryptococcus.* Species within genus *Cryptococcus* are responsible for invasive fungal infection, which cause Cryptococcosis and present substantial therapeutic challenges [12]. Consistent with our study, *Pseudomonas aeruginosa* has been reported to inhibit the growth of *Cryptococcus* species by producing antifungal molecules pyocyanin and an extracellular quorum-sensing signal 2-heptyl-3,4-dihydroxyquinoline [13]. Thus, such correlations need to be taken into consideration in clinical practice during the treatment for bacterial or fungal infections. 

Age and BMI have influence on the gut microbiome. In present study, there were three bacteria genera (*Pseudoflavonifractor, Oribacterium,* and *Flavonifractor*) positively correlated with age and one negatively correlated with age (*Mitsuokella*) (Supplementary Table S1). Two bacteria genera were positively correlated with BMI, *Xanthomonas* and *Curtobacterium*, meanwhile four were negatively correlated with BMI, including *Sphingomonas, Phascolarctobacterium, Holdemania,* and *Megamonas* (Supplementary Table S1). The correlation between age, BMI, and fungal genera was reported in our previous study, where *Altemaria* was found to be negatively correlated with age but no significant correlation was found between BMI and fungal genera [5].

There are several limitations of this study. First, due to the limit of sample availability, ITS and 16S rRNA sequencing were not performed in all enrolled patients with alcoholic hepatitis. Second, patients with chronic alcoholic hepatitis were lacking in our patient cohort. Third, the sample size in our study is small and correlations need to be validated in a larger and independent patient cohort. Finally, it is noteworthy that this correlation approach may not be representative of causal relationship in microbial ecology.

In conclusion, this study highlights the correlation between the gut bacteria and fungi, which needs to be taken into consideration in clinical practice. Further mechanistic studies of fungal and bacterial ecological interactions in alcohol-related liver disease are required.

## 4. Materials and Methods 

### 4.1. Patients

Non-alcoholic controls and patients with alcohol use disorder were recruited from an alcohol withdrawal unit in San Diego, USA, and Brussels, Belgium, where they followed a detoxification and rehabilitation program. Non-alcoholic controls consumed less than 20 g alcohol/day. Patients with alcohol use disorder fulfilled the Diagnostic and Statistical Manual of Mental Disorders, Fourth Edition (DSM IV) criteria. Patients were recruited with self-reported active alcohol consumption greater than 60 g/day. Patients were excluded if they have diabetes, inflammatory bowel disease, known liver disease of any other etiology, or clinically significant cardiovascular, pulmonary, or renal co-morbidities. During the 2 months preceding enrollment, non-alcoholic controls or patients with alcohol use disorder did not take immunosuppressive medication or antibiotics. Alcoholic hepatitis patients were enrolled in different medical centers in Europe and North America that were part of the InTeam Consortium (ClinicalTrials.gov identifier number: NCT02075918) between June 2014 and May 2018. Patients were enrolled when they were admitted to the hospital. Inclusion criteria for alcoholic hepatitis were: (1) active alcohol use (>50 g/day for men and >40 g/day for women) in the last 3 months, (2) aspartate aminotransferase (AST) > alanine aminotransferase (ALT) and total bilirubin >3 mg/dL in the past 3 months, and (3) liver biopsy and/or clinical picture consistent with alcoholic hepatitis. Patients who received antibiotics, proton pump inhibitors, and laxatives were included. Exclusion criteria were: (1) autoimmune liver disease (ANA > 1/320), (2) chronic viral hepatitis, (3) hepatocellular carcinoma, (4) complete portal vein thrombosis, (5) extrahepatic terminal disease, (6) pregnancy, and (7) lack of signed informed consent. Alcoholic hepatitis patients were recruited from 10 different medical centers in North America and Europe. The protocol was approved by the Ethics Committee of each participating center and written informed consent was obtained from each subject from each center.

### 4.2. Shotgun Metagenomic Analysis

DNA was extracted from stool samples collected from 9 non-alcoholic controls, 41 patients with alcohol use disorder, and 81 patients with alcoholic hepatitis. DNA extraction and library preparation were performed as described previously [14]. Shotgun metagenomics sequencing was performed on Illumina HiSeq 4000 generating 150 bp paired-end reads. KneadData version 0.7.2 was used for quality control. Metagenomic Phylogenetic Analysis 2 (MetaPhlAn2) version 2.7.7 was used for the profiling of the composition of the microbial community [15]. The HMP Unified Metabolic Analysis Network 2 (HUMAnN2) version 0.11.1 was used for the profiling of microbial pathways [16]. MetaCyc database was used for microbial pathway analysis [17]. Each of the HUMAnN2 abundance output was normalized into relative abundance (the counts for each sample sum to 100). Linear discriminant analysis (LDA) effect size (LEfSe) was used to find the pathways, which were different between control subjects, alcohol use disorder patients, and alcoholic hepatitis patients [18].

### 4.3. ITS Sequencing

Fungal DNA was extracted from 56 patients with alcoholic hepatitis, 15 patients with alcohol use disorder, and 10 non-alcoholic controls. For each sample, 1 g of zirconium oxide beads (0.5 mm diameter) was added and beaten for 30 s twice using a bead beater instrument (BioSpec Product Inc., Bartlesville, OK, USA), followed by a published DNA extraction protocol [5,19,20]. Internal transcribed spacer (ITS) sequencing targeting fungal ITS1 region was performed using Illumina MiSeq V2 kit, 300 cycles using primers. Primers, PCR conditions, and data processing were described in our previous study [5]. 

### 4.4. 16S rRNA Sequencing

16S rRNA PCR targeting the V4 region of the 16S rRNA gene was performed in 56 patients with alcoholic hepatitis, 15 patients with alcohol use disorder, and 10 non-alcoholic controls. These subjects were the same as the ones for ITS sequencing. DNA extraction and library construction were described previously [21]. Library was sequenced on Illumina MiSeq (Illumina) using V2 reagent chemistry, 500 cycles, and 250 bp paired-end reads. Furthermore, 16S sequence reads were analyzed using MOTHUR-based 16S rDNA analysis workflow as previously described [21]. 

### 4.5. Random Forest Model

We built random forest model to separate patients with alcoholic hepatitis from non-alcoholic controls and patients with alcohol use disorder using bacteria and fungi genera as detected by ITS sequencing and 16S rRNA sequencing. Extra-trees classifier was used to select 5 variables from bacteria or fungal genera. H_2_O platform (https://www.h2o.ai, accessed on 12 October 2020) was used to build random forest model. The dataset was split into training and test datasets (80:20). Stratified fivefold cross-validation was performed on the training set to choose the tuning parameters for the model.

### 4.6. Fungi–Bacteria Network in Alcoholic Hepatitis Patients

We examined the associations between fungi and bacteria genera in alcoholic hepatitis patients as detected by ITS sequencing and 16S rRNA sequencing. Genera were excluded if they were found in less than 5 patients. Sparse InversE Covariance estimation for Ecological Association and Statistical Inference (SpiecEasi) was used to estimate the association between fungi and bacteria [22]. Meinshausen and Bühlmann (MB) method was used as the neighborhood selection framework, which generates local conditional independence structure predictions while solving p regularized linear regression issues [23]. 

## Figures and Tables

**Table 1:** Subject characteristics for the metagenomic analysis.

	Non-Alcoholic Controls	Alcohol Use Disorder	Alcoholic Hepatitis	*p*-Value
Clinical parameter				
Total n	9	41	81	
Age (years), *n* = 130	51 (27–71)	44 (27–67)	51 (30–75)	0.016 *
Body mass index (BMI), kg/m^2^, *n* = 118	23 (19–29)	24 (18–37)	28 (19–48)	<0.001 *
Male gender, *n* (%), *n* = 130	7 (78)	34 (83)	54 (67)	0.186
Laboratory parameter				
Albumin (g/dL), *n* = 112		4.5 (2.2–5.2)	2.4 (1.3–4.1)	**<0.001**
Alkaline phosphatase (U/L), *n* = 113		68 (33–225)	179 (21–1153)	**<0.001**
ALT (U/L), *n* = 121		37 (9–184)	45 (15–216)	0.092
AST (U/L), *n* = 121		36 (15–283)	132 (41–406)	**<0.001**
Total bilirubin (mg/dl), *n* = 118		0.5 (0.2–1.5)	16.3 (2.5–38.6)	**<0.001**
GGT (U/L), *n* = 77		43 (4–1131)	188 (33–3632)	**<0.001**
Platelet counts (×10^9^/L), *n* = 115		222 (21–434)	123 (21–447)	**<0.001**
Prothrombin time, s, *n* = 65			22 (11–61)	
Creatinine (mg/dL), *n* = 118		0.8 (0.5–1.3)	0.8 (0.3–8.1)	0.791
Sodium (mEq/L), *n* = 79			133 (118–143)	
INR, *n* = 116		0.9 (0.8–1.3)	1.8 (1.0–3.7)	**<0.001**
FIB-4, *n* = 115		1.3 (0.4–21.4)	8.0 (1.4–66.3)	**<0.001**
FIB-4 > 3.25 (F3–F4), *n* (%)		6 (15)	71 (88)	

Note: Values are presented as median and range in parentheses. The total number of patients for which the respective data were available is indicated in the first column. In blank cells, patients from the respective group were not counted to missing numbers. Kruskal–Wallis test was used for three group comparisons. Tukey and Kramer (Nemenyi) post hoc test was used for pairwise comparisons. * Age (alcohol use disorder vs. non-alcoholic controls *p*-value: 0.650, alcoholic hepatitis vs. non-alcoholic controls *p*-value: 0.799, and alcoholic hepatitis vs. alcohol use disorder *p*-value < 0.012). * BMI (alcohol use disorder vs. non-alcoholic controls *p*-value: 0.996, alcoholic hepatitis vs. non-alcoholic controls *p*-value: 0.138, and alcoholic hepatitis vs. alcohol use disorder *p*-value: 0.001). Mann–Whitney test was used for two group comparison. Bold font indicates significance (*p*-value < 0.05). ALT, alanine aminotransferase; AST, aspartate aminotransferase; INR, international normalized ratio; GGT, gamma-glutamyl-transferase; BMI, body mass index.

**Table 2:** Characteristics of alcoholic hepatitis patients for the metagenomic analysis.

Treatment at Admission			Clinical Characteristics		
Steroids, *n* (%), *n* = 79		31 (39)	Model for end-stage liver disease (MELD), *n* = 79		24 (12–46)
Pentoxifylline, *n* (%), *n* = 65		6 (9)	MELD > 21, *n* (%), *n* = 79		62 (78)
Steroids and pentoxifylline, *n* (%), *n* = 65		1 (2)	30-day mortality rate, *n* (%), *n* = 76		8 (11)
Antibiotics, *n* (%), *n* = 79		18 (23)	90-day mortality rate, *n* (%), *n* = 56		13 (23)
Proton pump inhibitors, *n* (%), *n* = 42		5 (12)	Infection at admission, *n* (%), *n* = 68		13 (19)
Laxatives, *n* = 36		18 (50)			
					
**Histology**					
Liver biopsy available, *n* (%), *n* = 81		47 (58)	Bilirubinostasis, *n* (%), *n* = 44	0	14 (32)
Stage of fibrosis, *n* (%), *n* = 45	0	2 (5)		1	21 (48)
	1	0 (0)		2	2 (4)
	2	5 (11)		3	7 (16)
	3	6 (13)	Ballooning, *n* (%), *n* = 45	0	24 (53)
	4	32 (71)		1	21 (47)
Lobular fibrosis, *n* (%), *n* = 44	0	4 (9)	Giant mitochondria, *n* (%), *n* = 41	0	35 (85)
	1	5 (11)		1	6 (15)
	2	2 (5)	PMN infiltration, *n* (%), *n* = 45	0	10 (22)
	3	33 (75)		1	21 (47)
Pericellular fibrosis, *n* (%), *n* = 45	0	11 (24)		2	14 (31)
	1	34 (76)	Inflammatory grade, *n* (%), *n* = 46	0	11 (24)
Grade of steatosis, *n* (%), *n* = 46	1	19 (41)		1	26 (56)
	2	14 (31)		2	9 (20)
	3	13 (28)	Mallory bodies, *n* (%), *n* = 44	0	6 (14)
				1	38 (86)

Note: Clinical characteristics of 81 alcoholic hepatitis patients. Values are presented as median and range in parentheses. The total number of patients for which the respective data were available is indicated in the first column. In blank cells, patients from the respective group were not counted to missing numbers. Fibrosis stages: 0 no fibrosis, 1 portal fibrosis, 2 expansive periportal fibrosis, 3 bridging fibrosis, and 4 cirrhosis. Lobular fibrosis: 0 no fibrosis, 1 zone 3 (centrilobular) fibrosis, 2 zone 2 + 3 (midzonal) fibrosis, and 3 panlobular fibrosis. Pericellular fibrosis: 0 absent, 1 present. Steatosis: 1 mild <33%, 2 moderate <33–66%, and 3 marked >66%. Mallory bodies: 0 absent and 1 present. Bilirubinostasis: 0 no, 1 hepato-canalicular, 2 cholangiolar, and 3 both. Ballooning: 0 occasional hepatocellular, 1 marked hepatocellular, and 2 none present. Megamitochondria: 0 absent and 1 present. PMN infiltration: 0 no, 1 mild, and 2 severe. Inflammation: 0 no, 1 mild, and 2 severe. PMN, polymorphonuclear infiltration. MELD: model for end-stage liver disease.

## Data Availability

Raw 16S rRNA sequencing reads can be found in the National Center for Biotechnology Information (NCBI) SRA associated with Bioproject PRJNA525701. Fungal sequencing data can be found under BioProject PRJNA517994.

## Supplementary Materials

The following are available online at https://www.mdpi.com/2072-6651/13/2/143/s1, Table S1: Correlation between age, body mass index (BMI), and bacteria genera.

## References

[1] Thursby E., Juge N. (2017). Introduction to the Human Gut Microbiota. Biochem. J..

[2] Osna N.A., Donohue T.M., Kharbanda K.K. (2017). Alcoholic Liver Disease: Pathogenesis and Current Management. Alcohol. Res..

[3] Casafont Morencos F., de las Heras Castaño G., Martín Ramos L., López Arias M.J., Ledesma F., Pons Romero F. (1996). Small Bowel Bacterial Overgrowth in Patients with Alcoholic Cirrhosis. Dig. Dis. Sci..

[4] Lang S., Fairfied B., Gao B., Duan Y., Zhang X., Fouts D.E., Schnabl B. (2020). Changes in the Fecal Bacterial Microbiota Associated with Disease Severity in Alcoholic Hepatitis Patients. Gut Microbes.

[5] Lang S., Duan Y., Liu J., Torralba M.G., Kuelbs C., Ventura-Cots M., Abraldes J.G., Bosques-Padilla F., Verna E.C., Brown R.S. (2020). Intestinal Fungal Dysbiosis and Systemic Immune Response to Fungi in Patients with Alcoholic Hepatitis. Hepatology.

[6] Fan D., Coughlin L.A., Neubauer M.M., Kim J., Kim M.S., Zhan X., Simms-Waldrip T.R., Xie Y., Hooper L.V., Koh A.Y. (2015). Activation of HIF-1α and LL-37 by Commensal Bacteria Inhibits Candida Albicans Colonization. Nat. Med..

[7] Cuskin F., Lowe E.C., Temple M.J., Zhu Y., Cameron E., Pudlo N.A., Porter N.T., Urs K., Thompson A.J., Cartmell A. (2015). Human Gut Bacteroidetes Can Utilize Yeast Mannan through a Selfish Mechanism. Nature.

[8] Everard A., Matamoros S., Geurts L., Delzenne N.M., Cani P.D. (2014). Saccharomyces Boulardii Administration Changes Gut Microbiota and Reduces Hepatic Steatosis, Low-Grade Inflammation, and Fat Mass in Obese and Type 2 Diabetic Db/Db Mice. mBio.

[9] Cooper T.G. (1984). Allantoin Degradation by Saccharomyces Cerevisiae--a Model System for Gene Regulation and Metabolic Integration. Adv. Enzymol. Relat. Areas Mol. Biol..

[10] Wang M., Chen W.-Y., Zhang J., Gobejishvili L., Barve S.S., McClain C.J., Joshi-Barve S. (2020). Elevated Fructose and Uric Acid Through Aldose Reductase Contribute to Experimental and Human Alcoholic Liver Disease. Hepatology.

[11] Ben Haj Khalifa A., Moissenet D., Vu Thien H., Khedher M. (2011). Virulence factors in Pseudomonas aeruginosa: Mechanisms and modes of regulation. Ann. Biol. Clin..

[12] May R.C., Stone N.R.H., Wiesner D.L., Bicanic T., Nielsen K. (2016). Cryptococcus: From Environmental Saprophyte to Global Pathogen. Nat. Rev. Microbiol..

[13] Rella A., Yang M.W., Gruber J., Montagna M.T., Luberto C., Zhang Y.-M., Del Poeta M. (2012). Pseudomonas Aeruginosa Inhibits the Growth of Cryptococcus Species. Mycopathologia.

[14] Gao B., Emami A., Zhou R., Lang S., Duan Y., Wang Y., Jiang L., Loomba R., Brenner D., Stärkel P. (2020). Functional Microbial Responses to Alcohol Abstinence in Patients with Alcohol Use Disorder. Front. Physiol..

[15] Truong D.T., Franzosa E.A., Tickle T.L., Scholz M., Weingart G., Pasolli E., Tett A., Huttenhower C., Segata N. (2015). MetaPhlAn2 for Enhanced Metagenomic Taxonomic Profiling. Nat. Methods.

[16] Franzosa E.A., McIver L.J., Rahnavard G., Thompson L.R., Schirmer M., Weingart G., Lipson K.S., Knight R., Caporaso J.G., Segata N. (2018). Species-Level Functional Profiling of Metagenomes and Metatranscriptomes. Nat. Methods.

[17] Caspi R., Billington R., Keseler I.M., Kothari A., Krummenacker M., Midford P.E., Ong W.K., Paley S., Subhraveti P., Karp P.D. (2020). The MetaCyc Database of Metabolic Pathways and Enzymes—A 2019 Update. Nucleic Acids Res..

[18] Segata N., Izard J., Waldron L., Gevers D., Miropolsky L., Garrett W.S., Huttenhower C. (2011). Metagenomic Biomarker Discovery and Explanation. Genome Biol..

[19] Yang A.-M., Inamine T., Hochrath K., Chen P., Wang L., Llorente C., Bluemel S., Hartmann P., Xu J., Koyama Y. (2017). Intestinal Fungi Contribute to Development of Alcoholic Liver Disease. J. Clin. Investig..

[20] Iliev I.D., Funari V.A., Taylor K.D., Nguyen Q., Reyes C.N., Strom S.P., Brown J., Becker C.A., Fleshner P.R., Dubinsky M. (2012). Interactions between Commensal Fungi and the C-Type Lectin Receptor Dectin-1 Influence Colitis. Science.

[21] Duan Y., Llorente C., Lang S., Brandl K., Chu H., Jiang L., White R.C., Clarke T.H., Nguyen K., Torralba M. (2019). Bacteriophage Targeting of Gut Bacterium Attenuates Alcoholic Liver Disease. Nature.

[22] Kurtz Z.D., Müller C.L., Miraldi E.R., Littman D.R., Blaser M.J., Bonneau R.A. (2015). Sparse and Compositionally Robust Inference of Microbial Ecological Networks. PLoS Comput. Biol..

[23] Meinshausen N., Bühlmann P. (2006). High-Dimensional Graphs and Variable Selection with the Lasso. Ann. Stat..

