# Supplementary material for: Fungi–Bacteria Correlation in Alcoholic Hepatitis Patients
Source: Toxins (Basel). 2021 Feb 14;13(2):143. doi: 10.3390/toxins13020143 (PMC7917833; doi:10.3390/toxins13020143)
Supplement: Supplementary file 1 [file toxins-13-00143-s001.pdf]

Supplementary Materials: Fungi–Bacteria Correlation in Alcoholic Hepatitis Patients

Bei Gao, Xinlian Zhang and Bernd Schnabl

Table S1. Correlation between age, BMI and bacteria genera.

Metadata	Bacteria	r-value	*p*-value
age	*Pseudoflavonifractor*	0.279	0.018
age	*Oribacterium*	0.251	0.034
age	*Flavonifractor*	0.239	0.043
age	*Mitsuokella*	−0.263	0.026
BMI	*Xanthomonas*	0.258	0.035
BMI	*Curtobacterium*	0.25	0.041
BMI	*Sphingomonas*	−0.247	0.044
BMI	*Phascolarctobacterium*	−0.262	0.033
BMI	*Holdemania*	−0.272	0.026
BMI	*Megamonas*	−0.272	0.026
